# Work stress among older employees in Germany: Effects on health and retirement age

**DOI:** 10.1371/journal.pone.0211487

**Published:** 2019-02-04

**Authors:** Jana Mäcken

**Affiliations:** Institute of Sociology and Social Psychology, University of Cologne, Cologne, Germany; Universtiy of Düsseldorf, GERMANY

## Abstract

**Background:**

Policy makers in aging societies aim for the extension of work lives by increasing the official retirement age. Despite these efforts, many people stop working before reaching this retirement age. The main reason for early retirement is poor health. Health in turn is influenced by exposure to the work environment. Furthermore, health and work stress are influenced by education, which may lead to different effects for the lowly and the highly educated.

**Objective:**

This study examines the relationship between work stress and retirement age. It investigates whether this relationship is mediated by health and moderated by education. Three dimensions of health are taken into account: self-rated health (SRH), depressive symptoms, and high cardiovascular risk diseases (HCVR).

**Methods:**

A German subsample of the longitudinal Survey of Health, Aging and Retirement in Europe (SHARE) was linked with register data of the German Public Pension Scheme (SHARE-RV). The sample followed 302 individuals aged 50 to 65 years at baseline from 2004 to 2014. The data contains information on work stress, measured by job control and effort–reward–imbalance (ERI), health, and age of retirement. Multi-group structural equation modeling was applied to analyze the direct and indirect effects of work stress on retirement age via health. Work stress was lagged so that it temporally preceded health and retirement age.

**Results:**

Lower job control and poorer SRH lead to a lower retirement age. Health does not operate as a mediator in the relationship between work stress and retirement age. Education moderates the relationship between work stress and health: high ERI leads to better SRH and better physical health of higher educated persons. Low job control increases the risk of depressive symptoms for persons with less education.

**Conclusions:**

Improving stressful working conditions, particularly improving job control, can prolong the working lives of employees and postpone retirement.

## Introduction

Europe’s workforce is aging rapidly, especially in Germany. This demographic change requires policies that seek to extend working lives, for example, by increasing the statutory pension age and closing early retirement pathways. The aim of these reforms is to increase the labor participation of older workers to secure the long-term sustainability of the social security system. However, the actual retirement age in Germany in 2016 was 63.2 years and thus below the statutory age of 65 years [1]. Moreover, the premature exit from paid work has also been a serious concern for individuals and companies. For individuals, leaving paid work might increase the risk of financial and social problems, while companies face a skill shortage. This highlights the importance of understanding risk factors contributing to early retirement. Previous research has shown that one primary reason for early retirement is poor health [2–4]. Health in turn is influenced by exposure to the job environment, in which most persons spend a comparatively high proportion of their lifetime [5–7].

This longitudinal study investigates the complex relationship between work stress, health, and retirement age in Germany by asking whether work stress has a direct effect on retirement age or if health partially mediates this relationship. In addition, it examines whether effects vary, depending on the education levels of the employees, as health is affected by educational attainment.

Most studies analyzing the influence of work stress on retirement have measured the direct effects of work stress on retirement and controlled for health status. However, some studies have shown that work stress can influence an employee’s health status as well [5,8–10]. Only a few studies have analyzed how the effects of work stress via health have subsequently influenced retirement. These studies show that working conditions explain about 20% of the association between health and disability retirement in the Finnish context [11,12].

By doing so, this study advances previous research in several ways. First, the use of the structural equation modeling (SEM) creates the opportunity to estimate effect sizes for direct and indirect effects (mediation). Second, three different health measures are taken into account: self-rated health (SRH), depressive symptoms, and high cardiovascular risk diseases (HCVR). Third, the dependent variable, retirement age, is drawn out of register data, which reduces reporting bias [13]. Furthermore, reversed causality between work stress and health can be ruled out due to the use of longitudinal data in the SEM framework. Finally, this study gains insights into the mechanism in the German context, as previous research has been mostly carried out in Scandinavia [4]. The German case is particularly interesting as the labor market and pension reforms aiming to delay retirement have been effective and the employment of older workers has increased steeply [14]. Early retirement is possible at the age of 63 for persons with an insurance record of at least 35 years. However, the pension benefit will be reduced by a permanent deduction. Disability retirement is possible at the age of 63 without pension cuts and at the age of 60 with a deduction of 10.8%. On the other hand, it is possible to postpone retirement and to increase the pension benefit by 0.5% per every additional month worked.

Work stress can be best explained by the two internationally established theoretical models: the demand–control model [15] and the effort–reward–imbalance model (ERI) [16]. The first model, also known as the job strain model, identifies stressful work in terms of high demands in combination with low control. Different studies have shown that the control dimension seems to be more important than occupational demands for retirement intentions and disability retirement [17,18]. The second model claims that an imbalance between high efforts and low rewards affects health and retirement decisions. Rewards can be financial, e.g. promotion prospects, including job security, or emotional through recognition and appreciation [19]. The two models complement each other, with the first one focusing on work content, and the second highlighting violations of reciprocity exchanges. The latter implies that high efforts are perceived as not being adequately rewarded and a gratification crisis arising. Both models predict higher risks of several stress-related health outcomes and retirement intentions, e.g. early retirement and disability pension [10,12,20–23].

A strong predictor of retirement is *self-rated health* [2,3]. SRH is a commonly used generic health indicator, which is not necessarily related to a certain medical condition, but broadly reflects the different dimensions of health not covered by specific measures of illness or disease [11,24]. Self-rated health predicts many health outcomes, such as functional limitations [25], mortality [26], and disability retirement [11]. Another predictor is *depression*. Depression is the leading cause of disability worldwide and contributes significantly to the global burden of disease and costs [27]. Work stress can trigger depression, especially among older employees, given the significance of exposure time and lower adaptability of older workers to changing working conditions [10,28]. Several studies have shown that depressive symptoms lead to earlier retirement [4,29,30]. Finally, work stress not only affects mental health, but also harms physical health. In particular, *cardiovascular diseases* (CVDs) can be caused by work stress, as long-lasting stress increases the risk of hypertension, stroke, and heart attacks [9,31]. All three health outcomes—SRH, depression, and CVD—can be caused by work stress and thus may lead to a lower retirement age. A meta-analysis by van Rijn et al. (2014) compared the associations between these health outcomes and retirement. Because no studies for the effects of depression on early retirement existed, van Rijn et al. analyzed their effects on disability retirement. Their results showed that SRH was the strongest predictor of disability retirement, followed by chronic diseases and mental health, respectively. This is because SRH globally reflects the health-related quality of life instead of merely covering the physical or mental dimension [4].

In addition, the influence of work stress and health on retirement age likely varies between individuals. Less educated employees have a higher risk of poor health and early retirement [18,32]. Educational qualification is a main determinant as it provides resources and capabilities for employees that are required for successful labor market integration. Less-educated employees often have less influence over their effort and therefore lower motivation to stay at work compared with higher-educated employees with more challenging work and a higher influence level [33]. However, the lower-educated employees might not have the financial resources to retire early [32]. It can be expected that work stress leads to a lower retirement age and that health mediates this relationship at least partially. Furthermore, work stress in less-educated groups leads to a lower retirement age than in higher-educated groups.

## Methods

### Data and sample

The associations between work stress, health, and retirement age in Germany were investigated with the longitudinal Survey of Health, Aging and Retirement in Europe (SHARE). The survey collected data on health as well as the social and economic circumstances of participants aged 50+ years [34]. Starting with the first wave in 2004 and 2005 in 11 European countries and Israel, follow-ups were conducted biennially until 2015. In Germany, random sampling was based on regional registers, and a multi-stage design was applied. The first German sample in 2004 consisted of 3,008 respondents, and the longitudinal response rate across all waves was 77.6%, including respondents who recovered after missing a wave. In addition, SHARE-Germany offers the unique opportunity to link survey information with administrative records from the German pension scheme (SHARE-RV), including information on exact retirement dates on a monthly base [35]. This is restricted data, which can be retrieved through a separate application procedure. The last wave of the SHARE used was in 2013. Because SHARE-RV was only available until 2014, it was not possible to use any later waves of the SHARE. In addition, respondents had to give consent for record linkage, and the linkage was 47.5% (S1 Appendix).

Permissions to use and store SHARE and SHARE-RV data were obtained from the European Research Infrastructure Consortium (SHARE-ERIC) and the Research Data Center of the German Pension Insurance (FDZ-RV). Data was anonymized before they were accessed and combined using social security numbers (SSN) as a unique identifier. Respondents were asked for written consent during the regular SHARE interview to collect respondents’ SSN. Subsequently, FDZ-RV converted SSN into an anonymized code, which allows the researcher to combine data, but not to access the unique SSN. Ethical approval for the SHARE study and SHARE-RV was given by the Ethics Committee of the University of Mannheim and the Ethics council of the Max Planck Society.

The final sample of the present study consisted of respondents between 50 and 65 years at their first observation. To be included in the analysis, respondents had to be in paid work at the start of the observation and needed a minimum of two follow-ups. Self-employed persons and civil servants were excluded as they differed from employees and were not eligible for the German pension scheme and by that not part of the SHARE-RV. After data preparation, 302 respondents fulfilled these criteria (S1 Appendix). Robustness checks showed that demographics between the original sample and the final sample did not differ.

### Retirement age

The dependent variable, retirement age, drawn from the register data was calculated on a monthly base. Respondents were asked for consent to record linkage, and only respondents with insured activities were part of the administrative records. Record linkage was possible since the third wave, which had a linkage rate of 61%. Only nine respondents retired before 59 years and were treated as outliers and recoded to 59 years.

### Work stress

The independent variable, work stress, was measured with shortened versions of the original scales of the demand–control model [36] and the effort–reward–imbalance model [16]. Given the constraints of a multidisciplinary approach, the inclusion of the full questionnaire was not possible in SHARE [20]. Job strain was restricted to the control dimension because the predictive power of control by far exceeded the power of demand [18]. Based on the questions “I have little freedom to decide how I make my work” and “I have an opportunity to develop new skills” with answers ranging from 1- strongly agree to 4- strongly disagree, a sum index for low control was built [20]. The second question was reversed, so that both items were negative. The index ranges from 2–8 and a higher score indicates lower job control.

The effort–reward imbalance model was constructed as recommended by the developers [37] and was used in different studies based on previous SHARE data [18,20,38]. The effort–reward imbalance model was restricted to two items measuring effort and five items measuring reward. The ERI was defined by the ratio of the sum score of effort items (nominator) divided by the sum score of reward items (denominator) adjusted for the number of items [37]. A higher score showed an effort–reward–imbalance. Both models have been found to be valid [37,39] and have been associated with health and retirement in previous studies [18,20,38].

### Health

As health can be seen as a multidimensional concept, three different health measures were included as mediators. SRH was measured using the question, “Would you say your health is… 1- excellent, 2- very good, 3- good, 4- fair, or 5- poor.”

Depressive symptoms were measured using the EURO-D depression scale [40]. The scale consisted of 12 items measuring the number of depressive symptoms in general population surveys [20]. The scale ranged from 0 to12, whereby a higher value indicated more depressive symptoms. The EURO-D scale has been tested as a valid and consistent indicator of elevated levels of depressive symptoms in cross-European studies [40].

Based on the WHO criteria, high cardiovascular risk diseases (HCVRs) were considered to be a group of high risk factors, such as hypertension, diabetes, and high blood cholesterol, which increase the risk of disorders of the heart and blood vessels, including coronary heart disease and stroke [31,41]. SHARE respondents were asked if a doctor had told them that they had any of the named 14 conditions. HCVR was coded as a dummy into 1 if a respondent named one of the following four answers: 1. A heart attack, including myocardial infarction or coronary thrombosis or any other heart problem, including congestive heart failure; 2. High blood pressure or hypertension; 3. High blood cholesterol; and 4. A stroke or cerebral vascular disease.

### Moderator

Education was measured with the International Standard Classification of Education (ISCED-97) using ISCED-97 as a dummy, which is 1 when respondents have tertiary education, e.g. a university degree (5–6), and 0 otherwise (0–4).

### Statistical analysis

Multi-group structural equation modeling (SEM) was applied to analyze the direct and indirect effects of work stress on retirement age via health. The advantage of multi-group structural equation models is that they enable the possibility to estimate direct and indirect effects for less-educated people as well as higher-educated people. They also test whether differences between these two groups are significant. Estimation was done with maximum likelihood with missing values (mlmv). Additionally, a correlation between the two work stress indicators was assumed. Longitudinal data enabled the analysis of a causal path. Of five waves, the last two time points for each respondent before retirement were used. Retirement age was measured at t, the health measures on t-1 and work stress at t-2. The design was chosen for causality reasons, as the cause must temporarily proceed the outcome. Three different models for each health measure were estimated: Cross-lagged panel models for health and work stress were chosen first to rule out reversed causality. Second, a longitudinal SEM of work stress, health, and retirement age were chosen to analyze the mediating effect of health. Third, the model was stratified by education using the multi-group option in SEM. Robust standard errors were estimated. All analyses were carried out with STATA 14.0.

## Results

The mean retirement age of the respondents was 63 years. Less-educated employees retired earliest (Table 1). Low job control was the highest for less-educated individuals. ERI was higher among highly educated individuals. Respondents with less education reported poor SRH, depressive symptoms, and HCVR more often than people with higher education. P-values based on a t-test showed that differences in retirement age between less-educated people and highly educated people were significant.

**Table 1:** Sample characteristics at baseline. Means, standard deviations in parentheses. P-values based on t-test.

				Low Education		High Education		
	Range	Mean	SD	Mean	SD	Mean	SD	P-Value
		N = 302		N = 203		N = 99		
Retirement Age	59–65	62.96	2.03	62.83	2.05	63.22	1.98	0.06
Low Control	2–8	4.16	1.52	4.29	1.54	3.92	1.46	0.07
ERI	0.25–3.5	1.16	0.58	1.11	0.59	1.24	0.54	0.08
SRH	1–5	3.21	1.00	3.34	1.04	2.95	0.86	0.00
Depressive Symptoms	0–11	2.07	2.00	2.38	2.13	1.43	1.51	0.00
HCVR	0–1	0.59	0.49	0.71	0.46	0.29	0.46	0.06
Female	0–1	0.45	0.50	0.71	0.45	0.29	0.45	0.09

**Fig 1:**
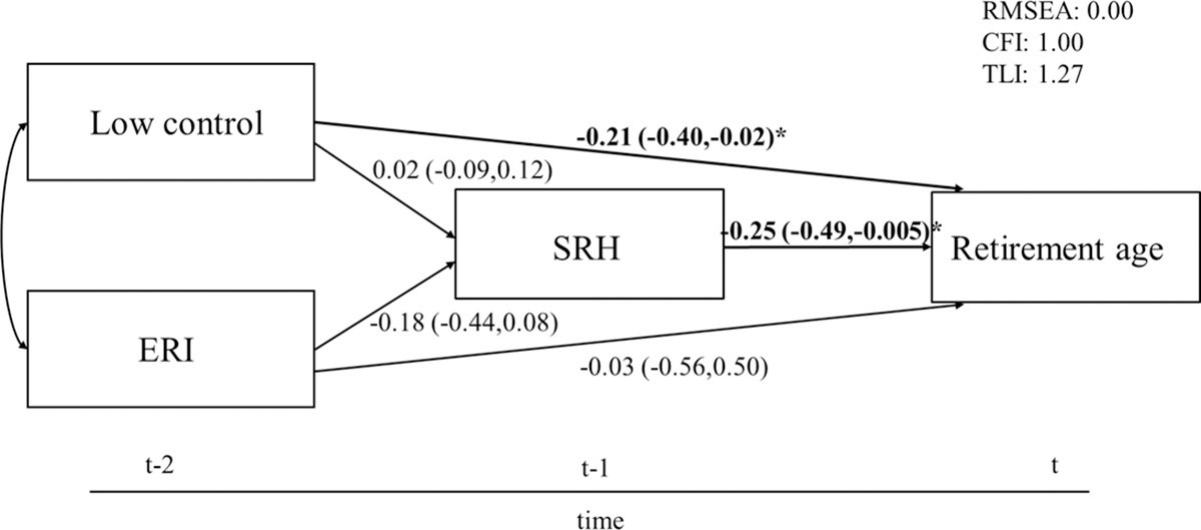
Structural equation model of the association between work stress, SRH and retirement age. 95% CIs in parentheses, adjusted for gender and education, N = 302. Levels of significance: *** p≤0.001; ** p≤0.01; * p≤0.05.

Results of the cross-lagged panel models showed that no reversed causality existed between work stress and the three health measures (S2 Appendix). The results of the structural equation model, which was adjusted for gender and education, showed no significant effects of low control and ERI on SRH (Fig 1). However, low control had a significant direct effect on retirement age. Respondents with low control had a significantly lower retirement age (B = -0.21, 95% CI-0.40;-0.02). In addition, poor SRH led to a significantly lower retirement age (B = -0.25 95% CI -0.49;-0.005). No indirect effects of work stress on retirement age were found. SRH did not mediate the association between work stress and retirement age. Fit indices showed a good model fit.

**Fig 2:**
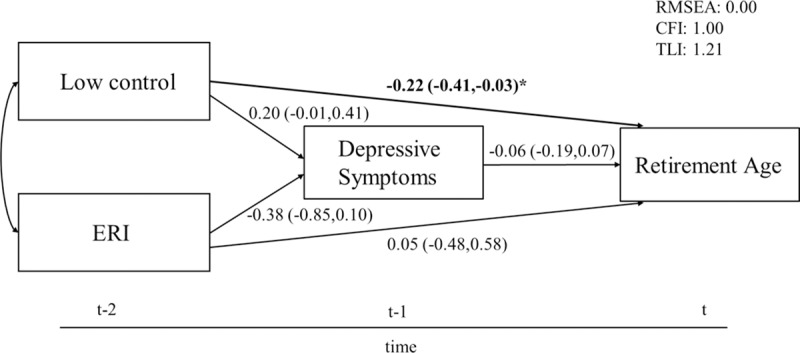
Structural equation model of the association between work stress, depressive symptoms and retirement age. 95% CIs in parentheses, adjusted for gender and education, N = 302. Levels of significance: *** p≤0.001; ** p≤0.01; * p≤0.05.

**Fig 3:**
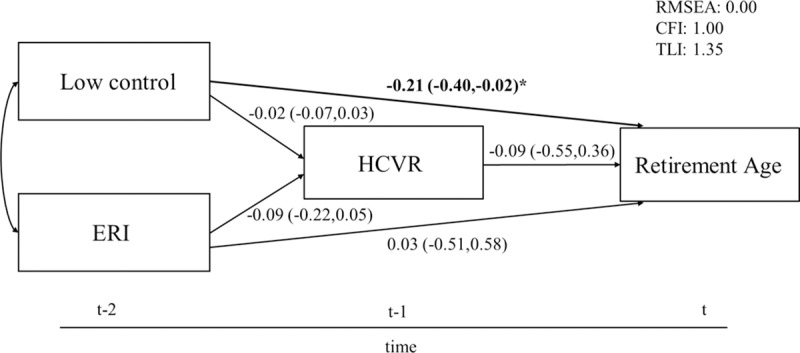
Structural equation model of the association between work stress, HCVR and retirement age. 95% CIs in parentheses, adjusted for gender and education, N = 302. Levels of significance: *** p≤0.001; ** p≤0.01; * p≤0.05.

Also, depressive symptoms (Fig 2) and HCVR (Fig 3) did not mediate the relationship between work stress and retirement age. Only low control had a significant direct effect on retirement age in both models (depressive symptoms: B = -0.22 95% CI -0.41;-0.03; HCVR: B = -0.21 95% CI -0.40;-0.02).

Multi-group models were estimated to examine whether effects differed based on educational level (Table 2). Educational differences were only significant in the association between work stress and health, but not in the retirement context. Higher ERI led to a significantly better SRH (B = -0.34, 95% CI -0.66;-0.01) for highly educated employees. For depression, low control increased depressive symptoms only for less-educated employees significantly (B = 0.32, 95% CI 0.02;0.62). In the event of HCVR, a higher ERI reduced the risk of an HCVR for highly educated employees (B = -0.22, 95% CI -0.39;-0.05).

**Table 2:** Direct effects of education on work stress, health, and retirement age, adjusted for sex, N = 302.

	Moderator: Education				
		Low Education		High Education	
		Coef.	95% CI	Coef.	95% CI	
On SRH	Low control	0.02	-0.13,0.17	0.03	-0.11,0.16	
	ERI	-0.10	-0.45,0.25	-0.34*	**-0.66,-0.01**	
On Retirement Age	Low control	-0.19	-0.44,0.05	-0.27	-0.61,-0.07	
	ERI	-0.20	-0.88,0.47	0.48	-0.41,1.37	
	SRH	-0.26	-0.53,0.02	-0.15	-0.64,0.34	
On Depression	Low control	0.32*	**0.02,0.62**	0.01	-0.20,0.21	
	ERI	-0.37	-1.02,0.28	-0.15	-0.74,0.43	
On Retirement Age	Low control	-0.19	-0.44,0.05	-0.27	-0.61,-0.07	
	ERI	-0.17	-0.83,0.50	0.48	-0.36,1.32	
	Depression	-0.05	-0.19,0.10	0.15	-0.43,0.13	
On HCVR	Low control	-0.01	-0.07,0.06	-0.01	-0.09,0.07	
	ERI	-0.01	-0.18,0.15	-0.22*	**-0.39,-0.05**	
On Retirement Age	Low control	-0.20	-0.44,0.03	-0.27	-0.61,-0.08	
	ERI	-0.17	-0.83,0.50	0.56	-0.32,1.44	
	HCVR	-0.12	-0.70,0.45	0.01	-0.77,0.79	

Levels of significance

*** p≤0.001

** p≤0.01

* p≤0.05

## Discussion

The aim of this study was to examine the relationship between work stress, health, and retirement age, based on educational level. The results show that health does not mediate the association between work stress and retirement age. Work stress in terms of low control has a direct effect on retirement age, showing that lower job control is associated with a lower retirement age. In contrast to previous research, effort–reward–imbalance has no effect on health and retirement age in Germany [18,38]. Additionally, poor SRH reduces retirement age, whereas depressive symptoms and HCVR do not. In line with previous research, this study shows that SRH seems to be a stronger predictor for early retirement than other conditions [4,11]. The results differ by education: Work stress affected health differently, depending on the level of education of the employee and the health measure. In the case of SRH, a higher ERI led to a better SRH for highly educated employees. Employees with a high level of education with a high ERI also had a lower probability of HCVR. In contrast, less-educated people with low job control had more depressive symptoms. No differences between educational level and retirement age were found.

This study contributes to previous research by showing that German employees tend to retire early when they perceive their job as stressful. Even though effect sizes in this study were small, among other factors, low job control and SRH can be assumed to play a role in the decision-making process on retirement [22]. Within this process, employees’ subjective assessment of their health status (SRH) matters more than the presence of depressive symptoms and high risk cardiovascular diseases. In line with previous research [4], this study shows that that self-rated health reflects a multidimensional concept of health and well-being, which goes beyond the absence of disease. Other important factors when considering retirement may be a higher preference for leisure time or family-related reasons, such as a retired spouse or grandchildren [3]. Further research, especially on the partner dyad, is needed on social determinants on retirement. Furthermore, in the present study, retirement age was calculated based on reliable register data, which has been rarely done in Germany. Labor market exits might occur before the actual retirement age, for example, when respondents become unemployed before they retire. This is not the case in the present study in which only employed respondents were considered who did not experience unemployment until retirement. As a result, the estimated work exit in the present study is a conservative estimation, and the gap between the statutory retirement age and work exit might be even larger when also considering episodes of unemployment. However, given the possibility of deriving the actual retirement age from register data, reporting bias has been minimized. Korbmacher (2014) showed that 40% of the German SHARE respondents misreported their retirement year with a deviation of three years on average [13]. The usage of register data is, hence, one contribution of this study. Additionally, reversed causality of work stress and health can be excluded based on the results of the cross-lagged panel models. Finally, this study closes a gap in previous research on retirement, health, and work stress by investigating the relationship in the German context, as most studies were carried out in Scandinavia [4]. Compared with the Scandinavian literature, no indirect effect of work stress on retirement was found in Germany [11,12]. In the Scandinavian context, depression and musculoskeletal diseases, determined by a physician, mediated the relationship between work stress and retirement and not a self-evaluation as in SHARE.

A limitation of the current study is the small sample size, which yields to low statistical power. Some effects might have been significant in a larger sample, for example the borderline-significant effects of low control on depression and SRH on retirement age for less-educated employees. Due to the small sample size, it was only possible to measure education binary. Respondents with tertiary education were put into the “highly educated group” and all other respondents became a part of the “less-educated” group. This led to subsuming a diverse group, including respondents with post-secondary education as well as primary education. The current sample cannot capture potential differences in work stress within the less-educated group. In addition, distinguishing old age pension from disability pension was not possible with the small sample, as only 5% of the respondents received a disability pension. Robustness checks were conducted by excluding respondents with disability pension, and the results did not change substantially. Moreover, stratifying instead of adjusting for gender showed no significant effects for women. This result may be mostly due to the smaller group size for women than for men. Nevertheless, it indicates that the associations between work stress and retirement age are stronger for men than for women. A replication of the study with a larger sample size could be a task for future research.

Germany is a special case regarding retirement, as the institutional background has changed much in the last 20 years. Specifically, manifold reforms aiming to delay retirement and active labor market measures helped to increase older workers’ employment [14,42]. In the current sample, 95% of the respondents were born before 1952 and thus had not been affected by these contextual changes. Additional analysis controlling for cohort showed no significant differences in the results.

The longitudinal design of this study covered five waves in total. A respondent’s retirement was modelled based on the respondent’s latest two waves before retirement, so that the independent variables temporarily preceded the dependent variable. Despite its advantages, this retrospective design yields the risk of a selection bias: Health and work stress were measured at older ages among those who retired later than among those who retired earlier, while risk of both poor health and retirement increase with age. Robustness checks controlling for age cohort did not reveal significantly different results, suggesting that such bias was limited. Furthermore, the sample was restricted to employees because civil servants and self-employed individuals were not part of the SHARE-RV. Results may, thus not be generalized to all occupational groups. This restriction is generally reasonable, as self-employed individuals have a greater degree of job autonomy and thereby likely retire later. Additionally, their lack of access to the public retirement scheme may postpone retirement [43].

The results of work stress on health differ from those of previous research, which showed that effort–reward–imbalance and low job control increase the risk of depression and cardiovascular diseases [5,7,9]. A possible explanation is the time lag between work stress and the health measures due to the longitudinal study design. As the SHARE is carried out biennially, the time lag may be too long as work stress can occur punctually and therefore is not captured adequately. In addition, the questions about work stress were ask broadly and not specific to a time frame. As a result, high scores only captured long-lasting, extreme stress. Analyzing work stress and the health measures cross-sectionally showed associations between work stress and health. Another explanation could be that some of the employees were not heavily exposed to work stress as they were working part time. While distinguishing between full-time and part- time work would have been desirable, this was not possible in a limited SEM. Moreover, the assessment of the two work stress measures was incomplete, as job strain was measured with two control dimension items only without any of the demand items. This detail increased the risk of underestimating the effects of work stress [20]. In addition, ERI was measured with a shortened scale, for example, excluding over-commitment for detecting coping with job demands. Sensitivity analyses have been done with the single dimensions of the ERI. In HCVR, only the effort dimension had a significant effect and in terms of SRH, the reward dimension was more important than effort. Not receiving the deserved recognition only affected the SRH of highly educated employees. Highly educated employees may be more overcommitted as they identify more strongly with their jobs. Moreover, coping mechanisms differ between highly educated and less-educated employees because highly educated individuals are more likely to possess helpful resources and are more adaptive [43,44]. Similarly, work stress is a subjective assessment, and respondents can become accustomed to stress or selecting themselves out of stressful jobs. Furthermore, the ERI is a measure for work stress among the whole workforce and not explicitly designed for older workers and specific educational groups. Some questions, e.g. job promotion prospects, may be less important for older workers or may even have the reverse effect. Receiving a promotion and new tasks shortly before retiring can even cause more stress. ERI might not be equally valid among special groups. Designing an ERI measure especially for older workers could be a task for future research. This idea may explain the effects of ERI on SRH and HCVR of highly educated people. Aside from these effects, less-educated employees may be more exposed to physical working conditions, such as noise or lifting heavy items, which are not captured in the present study. Instead, the ERI scale included a subjective assessment of the physical demands of the respondents’ jobs (e.g., “My job is physically demanding” is part of the ERI effort dimension). Data on objective conditions would have been desirable, and future research should include observational variables, for example, by linkage to a job-exposure matrix. However, SHARE did not offer this option.

Improving psychosocial working conditions can help to reduce early retirement beyond workers’ health status. In particular, improving job control potentially extends people’s work life, as it directly contributes to explaining low retirement ages in the present study. Policy makers and stakeholders, such as employers and trade unions, should closely monitor people’s work stress if they wish to prolong working lives and tackle the shortage of skilled professionals in times of demographic change.

## Supporting information

S1 Appendix Attrition in SHARE. (DOCX)

S2 Appendix Cross-lagged-panel models. (DOCX)
