# Supplementary material for: Work stress among older employees in Germany: Effects on health and retirement age
Source: PLoS One. 2019 Feb 4;14(2):e0211487. doi: 10.1371/journal.pone.0211487 (PMC6361437; doi:10.1371/journal.pone.0211487)
Supplement: S1 Appendix — (DOCX) [file pone.0211487.s001.docx]

| Attrition in SHARE | N | % |
| --- | --- | --- |
| SHARE-RV sample | 4148 | 100 |
| Remove respondents which participated only once | 3449 | 83,1 |
| Remove respondents which retired before first wave | 2606 | 62,8 |
| Remove respondents, which are not aged between 50 and 65 | 2440 | 58,8 |
| Remove respondents which had a transition to retirement but only one observation left | 1808 | 43,6 |
| Remove respondents if unemployed or homemaker at first observation | 1416 | 34,1 |
| Remove respondents without event (retire) during observation period | 302 | 7,3 |
|  |  |  |
