# Supplementary material for: Work stress among older employees in Germany: Effects on health and retirement age
Source: PLoS One. 2019 Feb 4;14(2):e0211487. doi: 10.1371/journal.pone.0211487 (PMC6361437; doi:10.1371/journal.pone.0211487)
Supplement: S2 Appendix — (DOCX) [file pone.0211487.s002.docx]

Figure 4: Cross-lagged-panel model ERI and SRH.


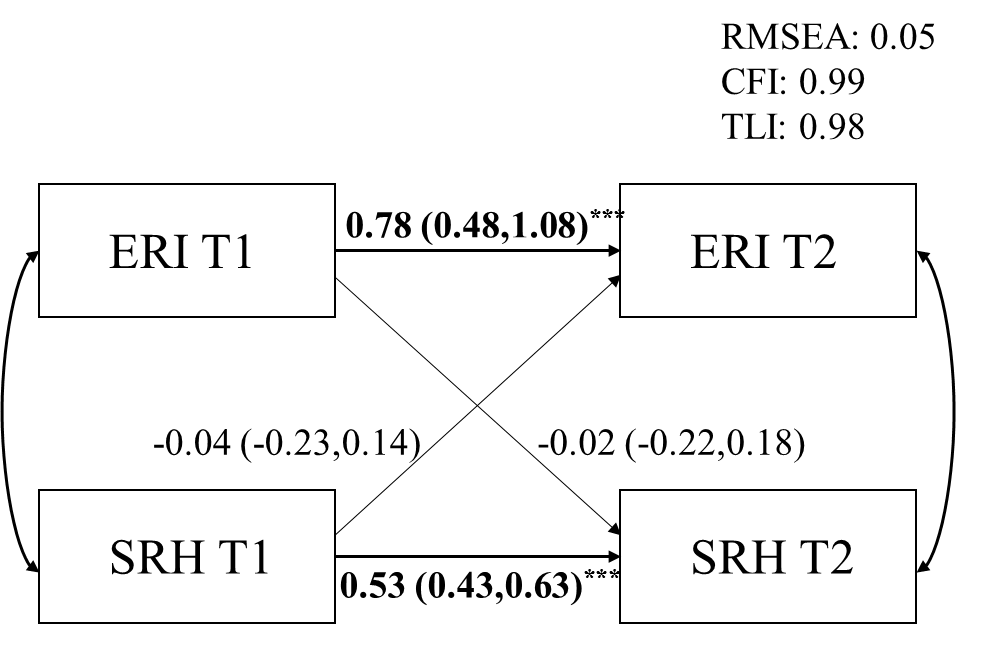


Levels of significance: *** p≤0.001; ** p≤0.01; * p≤0.05

Figure 5: Cross-lagged-panel model low control and SRH.


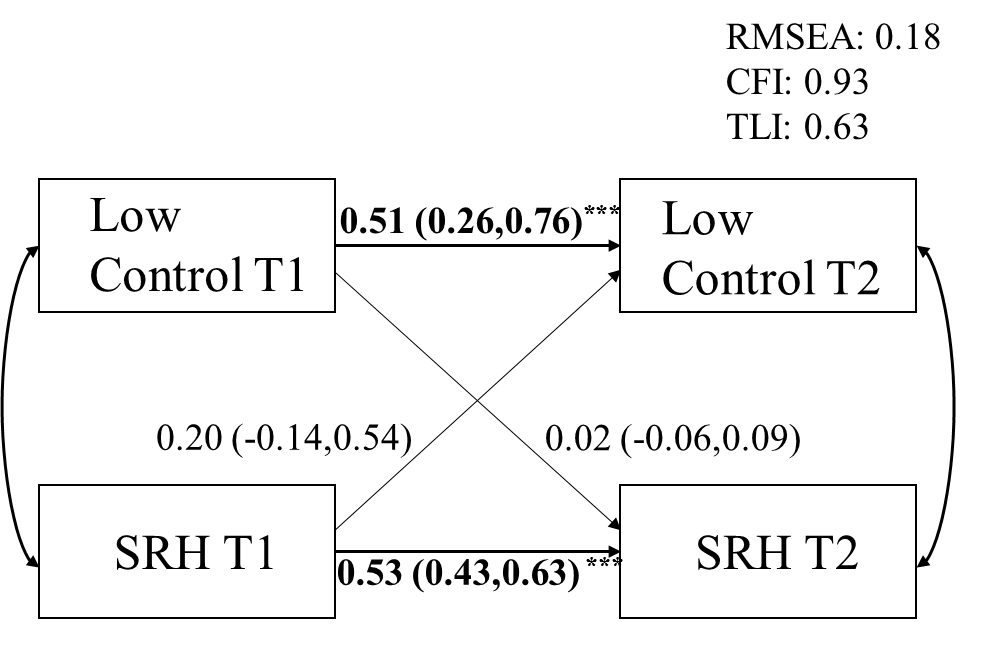


Levels of significance: *** p≤0.001; ** p≤0.01; * p≤0.05

Figure 6: Cross-lagged-panel model ERI and depressive symptoms.


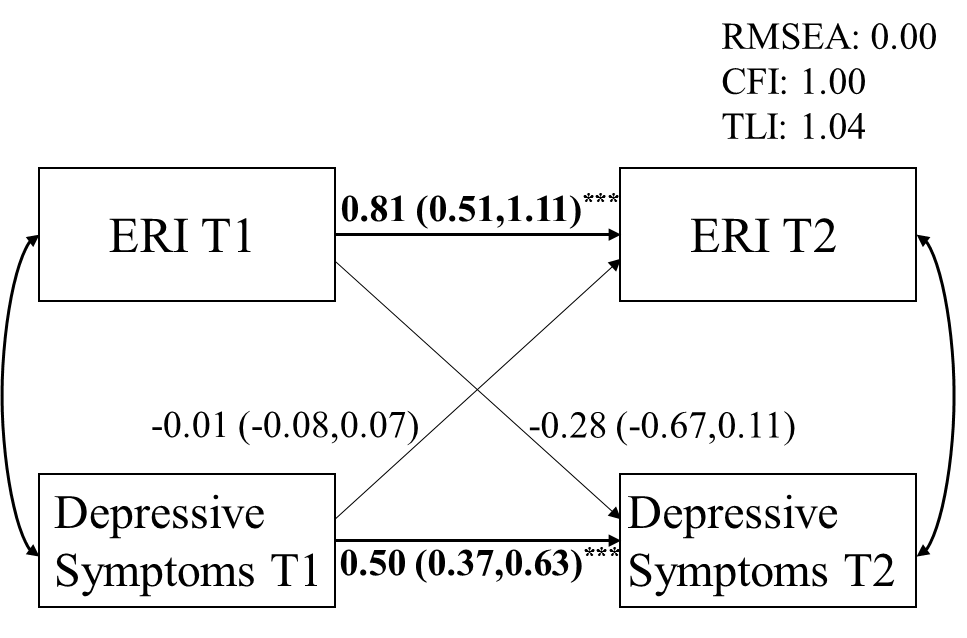


Levels of significance: *** p≤0.001; ** p≤0.01; * p≤0.05

Figure 7: Cross-lagged-panel model low control and depressive symptoms.


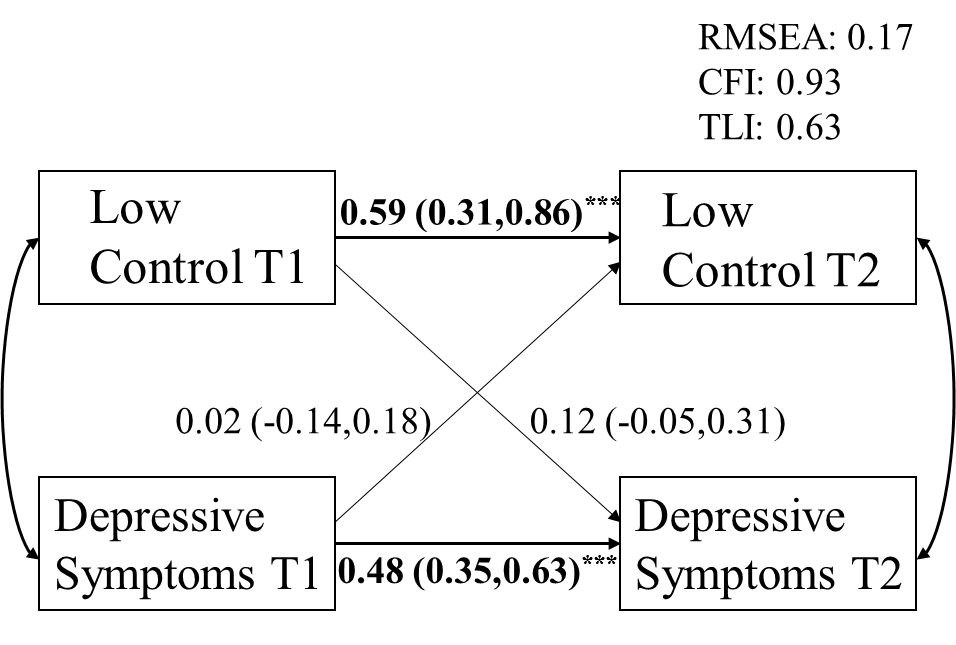


Levels of significance: *** p≤0.001; ** p≤0.01; * p≤0.05

Figure 8: Cross-lagged-panel model ERI and HCVR.


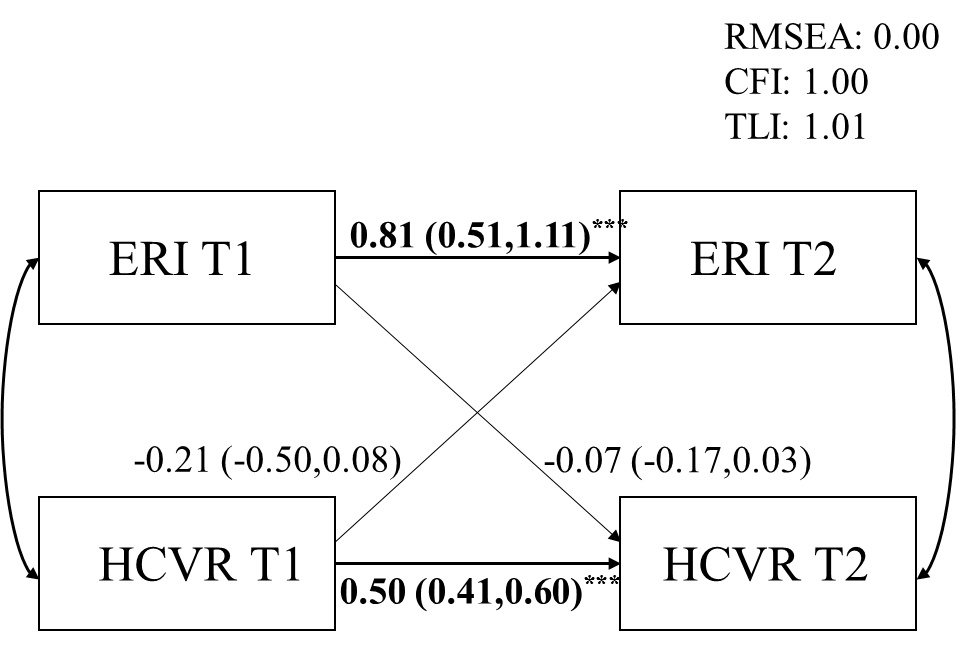


Levels of significance: *** p≤0.001; ** p≤0.01; * p≤0.05

Figure 9: Cross-lagged-panel model low control and HCVR.


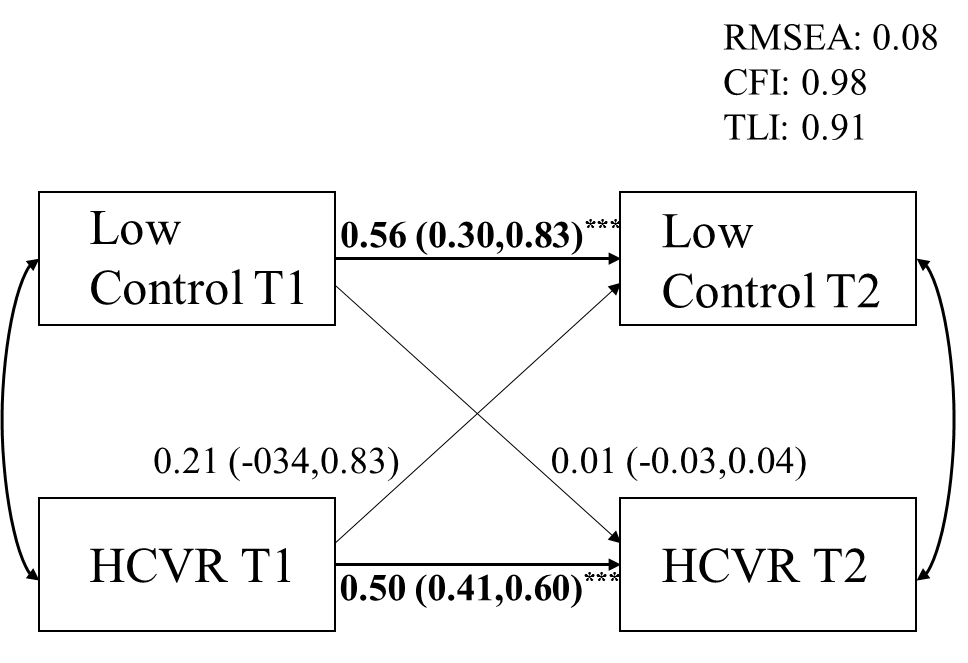


Levels of significance: *** p≤0.001; ** p≤0.01; * p≤0.05
